# Complete Genome Sequence of a *Staphylococcus epidermidis* Strain with Exceptional Antimicrobial Activity

**DOI:** 10.1128/genomeA.00004-17

**Published:** 2017-03-09

**Authors:** Simon B. Lassen, Hans B. Lomholt, Holger Brüggemann

**Affiliations:** Department of Biomedicine, Aarhus University, Aarhus, Denmark

## Abstract

*Staphylococcus epidermidis* is a Gram-positive bacterium that is prevalent on human skin. The species is associated with skin health, as well as with opportunistic infections. Here, we report the complete genome sequence of *S. epidermidis* 14.1.R1, isolated from human skin. In bacterial interference assays, the strain showed exceptional antimicrobial activity.

## GENOME ANNOUNCEMENT

*Staphylococcus epidermidis* is a species that constitutes a significant part of the human skin microbiota (1). It is mainly regarded as a health-beneficial organism, but strains of *S. epidermidis* can “accidently” cause opportunistic infections, such as those related to indwelling medical devices (2, 3). Multilocus sequence typing and genome sequencing studies have previously shown that *S. epidermidis* is a heterogeneous species with extended strain-level variation (1, 4).

A previous study investigated microbial interferences between members of the human skin microbiota; strains of *S. epidermidis* were isolated from human skin and their antimicrobial activities against *Propionibacterium acnes* were determined (5). One *S. epidermidis* strain, designated 14.1.R1, exhibited a broad anti–*P. acnes* activity. Initially, an Illumina short-read sequencing approach was carried out, which resulted in 131 assembled contigs (GenBank accession no. AGUC00000000.1) (5).

Here, we present the complete genome sequence of strain 14.1.R1. Genomic DNA of *S. epidermidis* was isolated using the MasterPure Gram-positive DNA purification kit (Epicentre). A genomic library was constructed and sequenced using a single-molecule real-time (SMRT) sequencing cell on a PacBio RS II machine at GATC (Germany); 87,774 sequence reads were obtained with an *N*_50_ read length of 23,465 bp and a mean read length of 15,770 bp. The assembly was done with HGAP3 (Hierarchical Genome Assembly Process 3), and resulted in four contigs with an average coverage of 372-fold. One contig corresponds to the circular chromosome (2,572,575 bp; G+C content 32.2%). The three other contigs represent extrachromosomal elements: two circular plasmids, pHOB1_14.1.R1 (24,769 bp; G+C content 30.8%) and pHOB2_14.1.R1 (9,323 bp; G+C content 28.3%), and a phage-like element, designated phage_14.1.R1 (18,659 bp; G+C content 32.9%). In addition to the sequence, information about all DNA base modifications of the genome of 14.1.R1 was obtained as well.

Gene prediction and annotation was performed using RAST (6), which predicted 2,582 genes on the chromosome, 22 genes on pHOB1, 13 genes on pHOB2, and 32 genes on the phage-like element. Phylogenetic analysis revealed that strain 14.1.R1 belongs to a phylogenetic clade that contains strains isolated from normal human skin and rodents, as well as endophytic strains isolated from rice seeds (5, 7). Genes associated with a polysaccharide-based biofilm (*ica* operon) are lacking from the genome of strain 14.1.R1. The chromosome harbors the genes for a type VII secretion system, located immediately upstream of a gene cluster encoding multiple nuclease toxins and their respective immunity factors (5). This system might be responsible for the broad antimicrobial activity of strain 14.1.R1 against *P. acnes*. In agreement, a nuclease toxin has recently been identified in *S. aureus* as a substrate of the type VII secretion system that can target competitor bacteria (8). Strain 14.1.R1 can be used as a model organism to study the role of the type VII secretion system in *S. epidermidis*.

### Accession number(s).

The genome sequence of strain 14.1.R1 was deposited in the DDBJ/EMBL/GenBank database under the accession numbers CP018841 to CP018844 (CP018841, phage HOB_14.1.R1; CP018842, chromosome 14.1.R1; CP018843, plasmid pHOB1_14.1.R1; and CP018844, plasmid pHOB2_14.1.R1).
